# Application of a genetic signature of late GU toxicity in SCIMITAR, a Post-op SBRT trial

**DOI:** 10.1016/j.ctro.2023.100594

**Published:** 2023-02-08

**Authors:** Amar U. Kishan, Nicholas Marco, Ting Martin Ma, Michael L. Steinberg, Ankush Sachdeva, Minsong Cao, Leslie K. Ballas, Emily Rietdorf, Donatello Telesca, Joanne B. Weidhaas

**Affiliations:** a University of California, Department of Radiation Oncology, Los Angeles, CA 90025, USA; b University of California, Department of Statistics, Los Angeles, CA 90025, USA; c University of California, Institute of Urologic Oncology, Los Angeles, CA 90025, USA; d Cedars Sinai, Department of Radiation Oncology, Los Angeles, CA 90048, USA

**Keywords:** SBRT, Genito-urinary toxicity, Germline biomarkers, PROSTOX

## Abstract

Predictors of genitourinary toxicity after post-prostatectomy radiotherapy remain elusive. A previously defined germline DNA signature (PROSTOX) has shown predictive ability for late grade ≥ 2 GU toxicity after intact prostate stereotactic body radiotherapy. We explore whether PROSTOX would predict toxicity among patients receiving post-prostatectomy SBRT on a phase II clinical trial.

## Highlights

• Genitourinary (GU) toxicity is a significant problem after short course radiotherapy (SBRT) for prostate cancer.
• Germline genetic biomarkers have been found to identify patients at significant increased risk of late GU toxicity.
• This genetic signature, PROSTOX, also identifies patients at increased risk of toxicity when SBRT is delivered after surgery.
• These findings validate the importance of PROSTOX in identifying patients at increased risk of GU toxicity after radiotherapy.

## 1 Introduction

Nearly-one third of patients who undergo radical prostatectomy will ultimately experience a biochemical recurrence [1]. However, post-operative radiotherapy is widely underutilized, in part due to concerns regarding post-treatment quality of life and toxicity [2]. In fact, predictors of genitourinary (GU) and gastrointestinal toxicity after post-operative radiotherapy remain largely elusive, though certain factors such as time to radiotherapy and age have been implicated [2]. There are data that suggest that genomic factors may be critical, with some studies suggesting that germline single nucleotide polymorphisms (SNPs) identified using genome wide association studies are associated with radiation toxicity [3]. However, these associations have been studied in the definitive radiotherapy context only, and focused on specific SNPs associated with solitary toxicity outcomes.

Our group recently applied a novel set of germline biomarkers, pathogenic variants disrupting microRNAs, and reported that a model based on 32 of these variants (PROSTOX) had excellent predictive ability for identifying patients at higher risk (greater than 55%) of developing late grade ≥ 2 GU toxicity after stereotactic body radiotherapy (SBRT) to the intact prostate [4].

We recently reported favorable early quality of life and toxicity outcomes from this phase II SCIMITAR trial (NCT03541850) of patients receiving SBRT to the prostate fossa [5]. While post-prostatectomy SBRT remains an experimental approach to be pursued only on a clinical trial, the prospective collection of germline DNA data for patients on the SCIMITAR trial allows for an evaluation of the proof-of-principle for whether a signature such as PROSTOX could predict toxicity in this context. In the present Short Communication, we report on the ability of the PROSTOX signature to predict late grade ≥ 2 GU toxicity after SBRT to the prostate fossa.

## 2 Materials and methods

The details of the SCIMITAR trial have been described previously [5]. In brief, 100 patients with an indication for postoperative radiotherapy after radical prostatectomy were enrolled between February 2018 to March 2021 and received SBRT directed to the prostatic fossa (30–34 Gy in 5 fractions), with or without pelvic nodal radiotherapy per investigator discretion (25 Gy in 5 fractions). The present analysis was restricted to the 61 patients receiving CT-guided radiotherapy, to be concordant with the original PROSTOX development cohort, which also utilized CT-guided SBRT. Clinical target volumes of the prostate bed and pelvic lymph node volume were delineated in accordance with the Radiation Therapy Oncology Group consensus guideline [6]. Each clinical target volume was expanded isotopically by 5 mm to form a corresponding PTV, which were then treated such that 95% of each PTV received prescription dose, unless doing so would lead to violations of OAR constraints (Supplementary Table 1). Late toxicity was defined as toxicity experienced 90 days or later after treatment. GU toxicity was based on CTCAE v 4.03. Per protocol, physician-scored toxicities were collected at baseline and at every 3 months for one year, and then every 6 months.

Development of the PROSTOX signature has been described previously [4]. DNA was extracted from  saliva samples collected from SCIMITAR patients at MiraDx, a CLIA lab performing PROSTOX as previously described [4], [7]. The assay was performed on each sample and a signature for late grade ≥ 2 GU toxicity after SBRT was created by using an Elastic Net model [8], utilizing 32 SNPs previously identified that are predictive of late GU toxicity. Performance metrics for the original cohort of patients receiving SBRT to the intact prostate were obtained by using leave-one-out cross-validation. Results from this signature were compared to the documented clinical toxicity for each tested patient from SCIMITAR. The analysis was limited to 59 patients who had at least 15 months of follow-up, as PROSTOX reports late GU toxicity. Clinical and demographic factors previously associated with late GU toxicity are tabulated in Supplementary Table 2. In this sub cohort**,** the median follow-up was 29.8 months (interquartile range, 24.0–39.5 months). Fifteen patients (24.6%) had late grade ≥ 2 GU toxicity, with the most common toxicity worsened/new incontinence, hematuria, and/or urgency (Supplementary Table 3).

Performance metrics for the new study were obtained to evaluate the performance of the PROSTOX signature on the SCIMITAR cohort of patients receiving CT-based treatments. An analysis of the toxicity free survival distribution for time to late GU toxicity was conducted by constructing Kaplan Meier curves [9] stratified by their predicted late GU toxicity outcomes. A corresponding log-rank test was conducted to compare toxicity-free survival between patients predicted to be at high-risk for late GU toxicity versus patients predicted to be low-risk.

## 3 Results

None of the historically identified predictors of late GU toxicity -- age, baseline pad use, time from surgery to SBRT, and baseline IPSS -- were significantly associated with late GU toxicity, all with p-values of greater than 0.2. On the other hand, PROSTOX performed well as a predictive variable. When compared with its performance in intact prostate SBRT, PROSTOX in the post-operative SBRT setting had a specificity of 0.95 (vs 0.92 for intact), a positive predictive value (PPV) of 0.80 (vs 0.65), a negative predictive value (NPV) of 0.86 (vs 0.96), and an AUC of 0.74 (vs 0.87). However, sensitivity was notably worse at 0.53 (vs 0.79), meaning that some patients that experienced toxicity were not considered to be high-risk individuals by the PROSTOX signature. However, patients predicted to be high-risk experienced toxicity 80% of the time. This, along with an AUC of 0.74, supports that the PROSTOX signature trained on late grade ≥ 2 GU toxicity after SBRT to the intact prostate is informative in predicting late grade ≥ 2 GU toxicity among patients receiving post-operative SBRT to the prostate fossa.

**Fig. 1:**
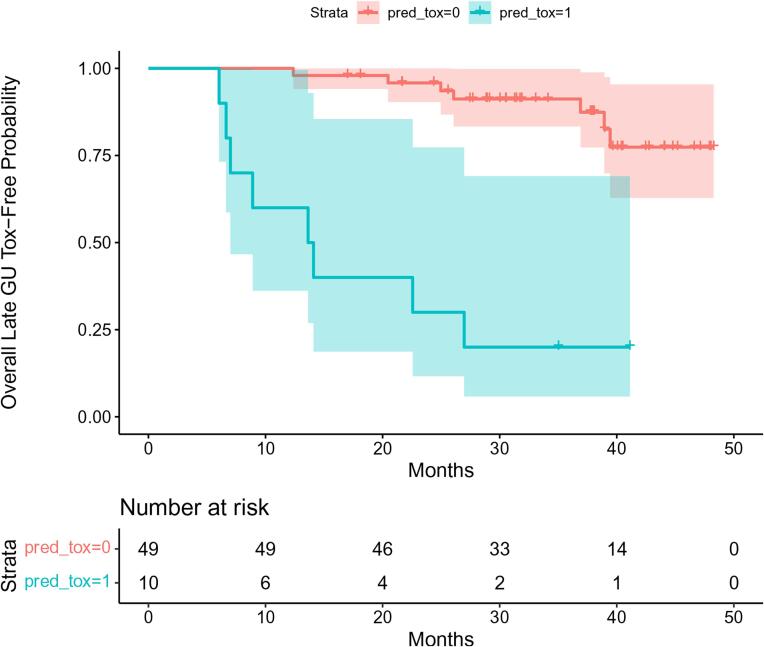
Late GU toxicity-free survival curves stratified by predicted late GU toxicity (<Grade 2) using the PROSTOX signature. The blue curve represents the estimated late GU toxicity-free survival curve of patients predicted to be at high-risk for late GU toxicity, while the red curve represents the late GU toxicity-free survival curve of patients predicted to be at low-risk for late GU toxicity.

A time until toxicity analysis was also conducted by estimating Kaplan Meier curves for patients predicted to be at high-risk versus low-risk for late GU toxicity by the PROSTOX signature (Fig. 1). A corresponding log-rank test of the two toxicity free survival curves found that those predicted to be at high-risk for late GU toxicity (Grade ≥ 2) in SCIMITAR using the PROSTOX signature had a significantly higher risk of toxicity (p = 0.0001, Fig. 1), further supporting the validity of PROSTOX to predict toxicity after CT-based SBRT even in the post-operative setting.

## 4 Conclusions

The high specificity, PPV, and NPV of the PROSTOX signature derived in the intact prostate SBRT setting when applied in this post-op SBRT setting suggests that the germline variant signature tested in PROSTOX may be a generalizable predictor of late GU toxicity after SBRT. However, the lower sensitivity supports the hypothesis that unique anatomic and functional differences (e.g., the presence of the prostatic urethra vs an anastomosis) might lead to differential toxicity profiles in these two settings.

It is important to note several important limitations of this study. First, it is a single center study and includes a relatively small cohort of patients, and therefore may not be broadly generalizable. Second, the outcome of interest for the signature was physician-scored toxicity, although patient-reported outcomes are of growing importance. Since patient-reported outcomes can be more nuanced and variable, for initial signature development (and thus for this validation study) the signature was “tuned” to predict physician-scored toxicity.

The predictive capacity of PROSTOX has now been prospectively studied in the GARUDA trial (NCT04624256); this signature is also being tested prospectively for all patients enrolled in the successor post-operative SBRT trial, EXCALIBUR (NCT04915508). These studies also include patient-reported outcomes and will thus contain important data to continue to validate the PROSTOX assay.

## Declaration of Competing Interest

The authors declare the following financial interests/personal relationships which may be considered as potential competing interests: Dr. Weidhaas is a co-founder of MiraDx, which holds intellectual property related to germline microRNA mutations as discussed in this manuscript. The other authors have no relevant conflicts of interest.
