# Supplementary material for: Application of a genetic signature of late GU toxicity in SCIMITAR, a Post-op SBRT trial
Source: Clin Transl Radiat Oncol. 2023 Feb 8;39:100594. doi: 10.1016/j.ctro.2023.100594 (PMC9984404; doi:10.1016/j.ctro.2023.100594)
Supplement: Supplementary data 1 [file mmc1.docx]

**Supplemental Tables**

**Supplemental Table 1**

| **Organ-at-Risk** | Volume | **Dose (cGy)** |
| --- | --- | --- |
| Anterior rectal wall (half) | Maximum point dose | No more than 105% of the prescription dose |
| Posterior rectal wall (half) | Maximum point dose | No more than 40% of the prescription dose (ideal)* |
| Rectal wall | <50% circumference | 24 Gy |
| Rectum | 45% | 27.5 Gy |
|  | 30% | 32.5 Gy |
|  | 25% | 33.75 Gy |
| Small intestine | Maximum point dose | 25 Gy (5.0 Gy per fraction) |
|  | Less than 10 cc | 20 Gy (4.0 Gy per fraction) |
| Bladder | Maximum point dose | No more than 105% of prescription dose |
|  | 35% | 32.5 Gy |
| Penile bulb | Maximum point dose | For tracking purposes only |
| Femoral heads | Less than 10 cc cumulative (both sides) | 20 Gy (4.0 Gy per fraction) |

**Supplemental Table 2**

|  | **Median (IQR)** |
| --- | --- |
| **Age** | 69.1 Years (65.6-71.3 Years) |
| **Time from RP to SBRT** | 23.8 Months (11.4-52.6 Months) |
| **Baseline IPSS** | 5 (2-9) |
| **Follow-up Time** | 29.8 (24.0-39.5 Months) |

**Supplemental Table 2:** Summary statistics of demographics and clinical measurements observed in the SCIMITAR cohort.

**Supplemental Table 3**

| **Toxicity Type** | **Grade ≥ 2 (% of Cohort)** |
| --- | --- |
| **Cystitis** | 3 (5.1%) |
| **Hematuria** | 9 (15.3%) |
| **Urinary Frequency** | 2 (3.4 %) |
| **Urinary Incontinence** | 11 (18.6%) |
| **Urinary Retention** | 1 (1.7%) |
| **Urinary Tract Infection** | 1 (1.7%) |
| **Urinary Urgency** | 7 (11.9%) |
| **Dysuria** | 2 (3.4%) |

**Supplemental Table 3:** Breakdown of the type of toxicity observed in the SCIMITAR cohort.
